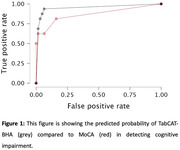# TabCAT Brain Health Assessment: Preliminary validation in a multicultural Israeli population

**DOI:** 10.1002/alz.091664

**Published:** 2025-01-03

**Authors:** Rafi Haddad, Elena Tsoy, David Tanne, Rawan Ayoub, Essam Shihada, Yarovinsky Natalya, Sabrina J Erlhoff, Tali Fisher, Judith Aharon‐Peretz, Rachel Ben‐Hayun, Victor Valcour, Katherine L Possin

**Affiliations:** ^1^ Global Brain Health Institute, University of California, San Francisco, San Francisco, CA USA; ^2^ Rambam Health Care Campus, Haifa Israel; ^3^ Memory and Aging Center, Weill Institute for Neurosciences, University of California, San Francisco, San Francisco, CA USA; ^4^ UCSF Memory and Aging Center, San Francisco, CA USA; ^5^ Stroke and Cognition Institute, Rambam Healthcare Campus, Haifa Israel; ^6^ Memory and Aging Center, University of California San Francisco, San Francisco, CA USA; ^7^ Rambam Healthcare Campus, Haifa Israel

## Abstract

**Background:**

The Israeli population, primarily comprised of Israeli Jews (74%) and Arabs (21%), is one of the most diverse aging societies around the world. The need for brief, accurate, and culturally appropriate cognitive measures in Israel is high, as they can facilitate early detection of cognitive disorders across clinical settings. We examined the discrimination accuracy and concurrent validity of the brief Tablet‐based Cognitive Assessment Tool (TabCAT) Brain Health Assessment (BHA) battery in a multicultural Israeli sample.

**Methods:**

Participants were 79 Hebrew and Arabic speaking older adults (age: 67±6; 58% female; education: 15±4). Clinically normal participants (n = 63) were community‐dwelling individuals with no self‐ or informant‐reported cognitive symptoms or functional decline. Cognitively impaired participants (n = 16) with diagnoses of mild cognitive impairment (n = 15) or dementia (n = 1) were recruited from a large neurology center. Diagnoses were based on the published clinical criteria, and all patients underwent comprehensive neurological and neuropsychological evaluations independent of study procedures. All participants completed the TabCAT‐BHA and the Montreal Cognitive Assessment (MoCA) in their primary language. Logistic regressions with ROC curves were used to examine discrimination accuracy, controlling for age, sex, education, and testing language. Concurrent validity was evaluated against the MoCA indices for the same domains.

**Results:**

The TabCAT‐BHA battery showed excellent discrimination accuracy (AUC = .99, sensitivity = .81, and specificity = .95) outperforming the MoCA (AUC = .94, sensitivity = .63, specificity = 0.97). Moderate associations were observed between TabCAT Favorites (associative memory) and MoCA Memory Index (r = .49, P < .001), and between TabCAT Match (executive functions) and MoCA Executive Index (r = .64, P < .001). Weaker associations were found between TabCAT Line Orientation (visuospatial skills) and MoCA Visuospatial Index (r = ‐.27, P = .02).

**Conclusions:**

Our preliminary findings support the validity of the 10‐minute TabCAT‐BHA battery in culturally diverse Israeli older adults. The battery exhibited excellent performance in detecting cognitive impairment in our sample outperforming a widely used brief cognitive assessment tool, the MoCA. Future studies, including development of Israel‐specific normative data and replication of these results in larger samples, are ongoing.